# Sexual Dysfunction before and after Cardiac Rehabilitation

**DOI:** 10.1155/2010/823060

**Published:** 2010-07-07

**Authors:** Jörg Schumann, Michael J. Zellweger, Marcello Di Valentino, Simone Piazzalonga, Andreas Hoffmann

**Affiliations:** Department of Cardiology, University Hospital of Basel, 4031 Basel, Switzerland

## Abstract

*Background*. The aim of this study was to assess sexual function before and after cardiac rehabilitation in relation to medical variables. *Methods*. Analysis of patients participating in a 12-week exercise-based outpatient cardiac rehabilitation program (OCR) between April 1999 and December 2007. Exercise capacity (ExC) and quality of life including sexual function were assessed before and after OCR. *Results*. Complete data were available in 896 male patients. No sexual activity at all was indicated by 23.1% at baseline and 21.8% after OCR, no problems with sexual activity by 40.8% at baseline and 38.6% after OCR. Patients showed an increase in specific problems (erectile dysfunction and lack of orgasm) from 18% to 23% (*P* < .0001) during OCR. We found the following independent positive and negative predictors of sexual problems after OCR: hyperlipidemia, age, CABG, baseline ExC and improvement of ExC, subjective physical and mental capacity, and sense of affiliation. *Conclusions*. Sexual dysfunction is present in over half of the patients undergoing OCR with no overall improvement during OCR. Age, CABG, low exercise capacity are independent predictors of sexual dysfunction after OCR.

## 1. Introduction

Sexual dysfunction is common in cardiovascular patients. The reported prevalence of erectile dysfunction (ED) reaches from 46% in men with coronary artery disease (CAD) [1] to 84% in men with congestive heart failure [2]. In another study 75% of male patients with CAD had problems achieving erection and 67% maintaining it [3].

A large proportion of patients do not return to normal sexual activity after a cardiac event, acute myocardial infarction (AMI), percutaneous coronary intervention (PCI), coronary artery bypass graft (CABG), or valvular surgery. Age, medication, and cardiovascular risk factors such as diabetes, hypertension, dyslipidemia, and smoking have been shown to be contributing to impaired sexual function or ED. In patients with sexual dysfunction, perception of well-being and self-esteem is generally depressed and contributes to worsen quality of life (QoL) [4–8]. 

Cardiac rehabilitation programs are important regarding education for secondary prevention in patients with heart disease and have been shown to improve functional capacity as well as quality of life and morbidity and mortality [9, 10]. 

Quality of life is defined as a person's subjective perception about his or her position in life in relation to the culture and the value system the person is living in and about the person's expectations, aims, and wishes (WHO 1997). This multidimensional concept is influenced by physical health, mental state, social relations, and personal beliefs. Sexual function is an important component of cardiac patients quality of life and overall well-being as has been shown earlier in [11–15]. Therefore we were interested in the effect of a short-time rehabilitation program on these parameters. Few trials have addressed the topic of sexual function in cardiac rehabilitation patients [16, 17] and its relation to exercise training [11]. One study compared different intensities of outpatient cardiac rehabilitation and their effect on QoL [18]. To our knowledge there is no data available on the course of sexual dysfunction during outpatient cardiac rehabilitation programs in general and about the effect of improved exercise capacity on sexual function in particular.

The aim of this study was to assess sexual function before and after cardiac rehabilitation in relation to various medical variables with special focus on exercise capacity and quality of life.

## 2. Methods

### 2.1. Participants

A total of 2085 consecutive patients (84.9% males, 15.1% females) were enrolled in an outpatient cardiac rehabilitation program (OCR) between March 1999 and December 2007. We included all patients assigned to our program except those with an LVEF of <30% and/or exercise capacity of <50 Watt who were assigned to a special CHF-program. There were no other prespecified inclusion or exclusion criteria. We analysed data from all 896 male patients who had exercise tests both at entry and at the end of OCR and who also had answered questionnaires about sexual function and quality of life at both points in time.

### 2.2. Measurements

Detailed *medical data* (AMI, PCI, CABG, cardiovascular risk factors, and medication) were available in all patients. Left ventricular ejection fraction (LVEF) was measured at least once by ventriculography, echocardiography, scintigraphy, or MRI. At the beginning of the OCR program all patients underwent a symptom-limited bicycle ergometer test to assess maximal exercise capacity using a ramp protocol targeted to an exercise duration between 6 and 12 minutes. Exercise capacity was expressed as maximal workload in watts (W). The electrocardiogram and blood pressure values were monitored during the test and the recovery phase. Exercise tests were stopped at the following end-points: subjective exhaustion, typical severe angina, ST-segment depression ≥2 mm, relevant arrhythmia, systolic hypertension >250 mm Hg, or a drop in systolic blood pressure of more than 10 mmHg below the resting value. 

Additionally we assessed *quality of life parameters* using a standardized questionnaire evaluating different aspects of the condition and feeling of the patients (**PLC** ( Profil der Lebensqualität Chronisch Kranker)) [19]. The PLC questionnaire was developed to transport an effective instrument of assessing QoL into German language [20]. The PLC has been tested [21–26] and validated [27] in various studies. It has been used in other studies [28] and has been translated into Spanish and evaluated in Spanish trials as well [29, 30].

This generic questionnaire was created to assess time-related changes in health for most of the chronic diseases and was selected for this study because of the heterogeneous nature of the patient cohort, its broad range of aspects, and the fact that it represents an instrument specifically developed for a German-speaking cohort. The aim of this self-assessment application is the quantitative acquisition of changes over time. The coremodule contains 40 Likert-scaled items scored from 0 (very bad) to 4 (very good) which cover the following aspects of quality of life within six scales: *subjective physical and mental performance* (**scale 1**), *ability to have pleasure and relaxation *(**scale 2**), *positive mood* (**scale 3**), *negative mood* (**scale 4**), *ability to relate/contact/approach (* **scale 5**), *and sense of affiliation* (**scale 6**).

This measuring system contains a high sensitivity for changes. In more detail scale 1 covers aspects of physical and mental functional capability in private and professional daily routine. Scale 2 refers to individual capability of mental regeneration including questions about quality of sleep, appetite and the ability to relax, have pleasure and compensate frustration and anger. Scale 3 addresses important aspects of positive temper like concentration, balance and confidence. In opposite scale 4 covers aspects of negative temper like depressiveness, excitability, hopelessness and threat. The aspect of social competence is covered in *scale 5* which evaluates the ability of making contacts and opening to as well as communicating with other people. Scale 6 covers aspects of social and emotional support like closeness, care, love and help. All these topics define the term “quality of life”.

*Sexual function* was assessed before and after OCR using *5 specific items included in the PLC questionnaire*: problems with or change of sexual activity, decrease in libido, problems due to exertion during sexual intercourse, problems with ED or orgasm, and no activity at all. These self-assessment questions could be answered with YES or NO. The terms “sexual activity” and “intercourse” were used as a generic term that was not further specified. The PLC questionnaire has proven its objectivity and reliability and has been validated for a variety of disease states, including CHD [11]. In psychometric statistics reliability can be measured by two different approaches: the internal consistency (reliability coefficient Cronbach's Alpha) and the test-retest reliability. The Cronbach's Alpha for the PLC scales is about *r* = 0.80 in average which is a good value.

### 2.3. Rehabilitation Program

The OCR of the Department of Cardiology of the University of Basel has been described previously in [28]. It is an ambulatory rehabilitation program for patients with coronary artery disease (prior myocardial infarction or angina pectoris with or without revascularization), valvular heart disease, previous cardiac surgery, or congestive heart failure consisting of prescribed and supervised exercise, relaxation, education, and counselling. The program is divided into a build-up and consolidation phase. The build-up phase consists of four weeks of intense rehabilitation with daily activities for about three hours per day. In the consolidation phase which lasts for another 8 weeks, patients have physical activities three times a week for approximately two hours each. Physical activities consist of endurance training on bicycle ergometers or treadmills at 60–80% of the maximal heart rate calculated at the baseline exercise test, of outdoor walking in topographically different areas, and of strength and coordination training. Approximately 30% of all patients after cardiac surgery or interventional revascularization at our institution are enrolled in the local OCR (approximately 290 pts. per year in build-up phase and 170 pts. per year in consolidation phase) whereas 40% participate in inpatient programs at specialized institutions and another 30% do not have any organized rehabilitation. Patients were referred to OCR based on their preferences or on those of their physicians.

### 2.4. Statistical Analysis

All continuous variables are described as mean ±SD for the equally distributed variables and as median for the nonequally distributed variables. Categorical variables are described as percent of the patient population. Comparisons between patient groups were performed using the (in)-dependent-samples T-test, the Mann Whitney U test, and the Wilcoxon test for continuous variables and an *χ* ^2^ test or McNemar's test for the unpaired and paired categorical variables, respectively. A *P*-value <.05 was considered statistically significant.

The independent predictors of sexual dysfunction were assessed using a binary logistic regression model. The inclusion of variables was based on univariate significance of variables during the course of rehabilitation and clinical judgement. A forward stepwise method was applied using a *P*-value <.05 for inclusion. SPSS version 16 was used for these analyses.

## 3. Results

Complete data including two questionnaires and exercise tests were available in 896 male patients (43% of the total population). Reasons for incomplete data were language deficits due to a migration background (27%), unwillingness to fill in repeated questionnaires (16%), early dropouts (6%), or otherwise incomplete data (8%). Baseline characteristics and exercise test results are summarized in Tables 1 and 2, respectively. Mean age was 62 years, 65% had PCI, which in about two thirds of all cases was performed as acute-PCI within the first few hours of an acute coronary syndrome, 29% had coronary artery bypass surgery (CABG), and 6% had valvular surgery. The data show a middle-aged population with an average risk profile and good functional capacity, which increases towards age-and-gender specific target values after OCR.

Details of responses to questions about sexual activity are given in Table 3(a). No sexual activity at all was indicated by 23.1% of patients at baseline and 21.8% after OCR, respectively. No problems with sexual activity were indicated by 40.8% at baseline and 38.6% after OCR, respectively. Patients showed an increase in specific problems (erectile dysfunction and orgasm) during OCR from 18 to 23% (*P* < .0001). Libido and problems due to exertion during intercourse did not differ significantly.

There was no overall improvement in sexual function during OCR. During OCR only a small number of patients changed regarding their sexual function (Table 3(b)).

We analysed the influence of LVEF, BMI, baseline and followup exercise capacity, and the increase in exercise capacity on sexual parameters (as defined in questionnaires).

The median value of LVEF was 60%. Patients with a LVEF >60% showed a trend to less “problems because of exertion during intercourse” at baseline (4.9% versus 7.9%; *P* = .091) and after OCR (4.9% versus 8.1%; *P* = .069).

**Figure 1:**
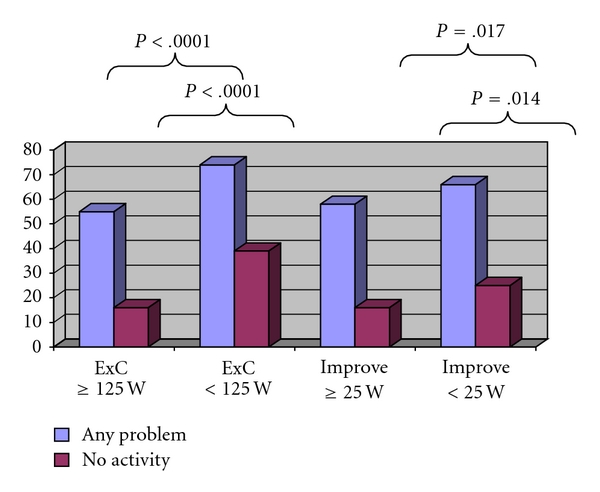
Percent of patients after OCR having any sexual problems (bright bars) or indicating sexual inactivity (dark bars) in subgroups of patients according to exercise capacity (left-sided pairs columns) and improvement of exercise capacity during OCR (right-sided pairs of columns) ExC: exercise capacity; improve: improvement in exercise capacity. OCR: outpatient cardiac rehabilitation.

The median value for exercise capacity was 125 Watt. Patients with an exercise capacity of ≥125 Watt at baseline had less problems with sexual function (any problems: 55% versus 74%; *P* < .0001) and had more sexual activity (no activity: 16% versus 39%; *P* < .0001) (Figure 1).

The median improvement of exercise capacity during OCR was 25 Watt. The degree of improvement in exercise capacity during OCR correlated with an improvement in sexual function. When compared to those with a lower than median improvement, patients with an improvement of ≥25 Watt had less problems with sexual activity and more sexual activity after OCR (any problems: 58% versus 66%; *P* = .009; no activity: 16% versus 25%; *P* = .002) (Figure 1).

Table 4 lists patient characteristics that predict having any problems with sexual activity after OCR.

In this univariate analysis age, CABG, hypertension, hyperlipidemia, and diabetes turned out to be predictors of having sexual problems after OCR whereas an exercise capacity of more than 125 Watt and an improvement of exercise capacity of at least 25 Watt predicted having no problems after OCR. All PLC scales turned out to be predictors of sexual function after OCR as well. In contrast PCI, BMI, family history, and smoking had no influence on this. Interestingly, there was no difference in patients taking betablockers compared to those without this medication. We did not further analyze other medications.

In a multivariate regression analysis we found independent positive and negative predictors of sexual problems after OCR as listed in Table 5(a).

When we included the baseline PLC scales in the multivariate analysis, Scales 1 (subjective physical and mental performance) and 6 (sense of affiliation) turned out to be additional independent predictors of having problems with sexual function after OCR whereas exercise capacity, and its improvement dropped out (Table 5(b)).

## 4. Discussion

Cardiac rehabilitation programs have been shown to reduce morbidity and mortality in cardiovascular patients and to improve quality of life [9, 10]. These metaanalyses identified exercise therapy to be a crucial element of cardiac rehabilitation. OCR programs consist of a multimodal approach addressing risk factor education, lifestyle modifications such as diet and exercise behaviour, and optimization of drug therapy. As far as we know no data are available about the course of sexual dysfunction during a cardiac rehabilitation program with special focus on its relation to exercise capacity.

In our study sexual dysfunction was present in over half of the patients undergoing outpatient cardiac rehabilitation with no overall improvement during OCR. The prevalence of sexual problems in our population is comparable to the data in literature [1, 2]. Patients showed a significant increase in specific problems like ED and orgasm during OCR.

In our population the mean LVEF was nearly normal. One possible explanation is a timely treatment by revascularisation of acute coronary syndromes avoiding significant myocardial damage in most cases. In addition patients after big infarcts tend to be referred preferentially to inpatient rehabilitation programs.

We identified age, CABG, hypertension, hyperlipidemia, diabetes, exercise capacity and its improvement as independent predictors of sexual dysfunction after OCR. This is plausible as these factors are markers of a more severe disease state.

The mean value of exercise capacity was 125 Watt what seems to be a surprisingly high value when considering that 24% had recent CABG and 9% valvular surgery. However there was a wide range of values (minimum 50 W, maximum 200 W; SD ± 37 Watt) accounting for this.

Interestingly betablockers did not influence sexual function in this population. However since 90% of the patients were on these drugs this might weaken the variable to become discerning. We suppose that the underlying disease as well as the drastic impact of an acute coronary event already causes a relevant impairment in sexual function and that betablockers in this setting do not have a significant additional effect. Similarly, in a study in cardiac rehabilitation, patients on Atenolol did not show a higher incidence of ED [16]. Otherwise this finding might be owed to “newer-generation” that is, selective betablockers with a possible neutral effect on ED. Indeed Metoprolol was used in a majority of patients included in this study.

Smoking, somewhat unexpectedly, did not appear as a predictor of ED. Paradoxically in another study where penile arterial blood flow was measured arterial insufficiency was least likely to occur in the smoking group [31]. Other variables seem to be more potent predictors which may have been underestimated in previous studies [32–34].

Whereas age and CABG have already been known as predictors of sexual dysfunction [4–8] the identification of exercise capacity and its improvement is new in this context. Good or rather higher-than-average physical fitness as well as an improvement thereof can reduce the risk of having sexual problems after OCR. It might be criticized that both patients after PCI and CABG have been merged and should have been analysed separately at least because of differences in exercise capacity. We accounted for this by performing a multivariate analysis that showed exercise capacity and its improvement to be independent predictors.

There was also a significant relation between parameters of quality of life and sexual dysfunction in this population. Not surprisingly, all aspects were independent predictors of sexual function after OCR, particularly PLC scales 1 (subjective physical and mental performance) and 6 (sense of affiliation). Therefore it seems that exercise capacity and its improvement are only surrogate markers and that subjective variables are even more predictive for problems with sexual activity or said in simple terms: “if you feel powerful you generally are.” Our findings point at the important role of self-motivation for physical activity not only for the time course of the OCR. In other words, more training leads to higher exercise capacity and self-confidence which predicts a better sexual function.

Preliminary data on the effectiveness of a structured approach with group therapy during rehabilitation showed a marked improvement of overall sexual activity as compared to usual care [35].

## 5. Conclusion

Sexual dysfunction is present in over half of the patients undergoing outpatient cardiac rehabilitation and—unlike physical exercise capacity and parameters of quality of life—does not improve during OCR. Sexual dysfunction is not related to ventricular function but rather to global exercise capacity and its improvement as well as to subjective physical and mental performance and sense of affiliation. Age and CABG are further independent predictors of sexual function after OCR. Thus sexual function seems to reflect overall well-being. A focus on residual capacities rather than on deficits might help to put patients at ease with themselves and improve sexual dysfunction.

## 6. Limitations

Our study has several limitations. The population is heterogeneous regarding the underlying diagnosis and indication for OCR. We analysed patients with CAD (AMI, PCI, and CABG) as well as patients after valvular surgery. The results may not be valid for all the subpopulations. However we suppose that especially for the majority of CAD patients the results would be similar.

There seems to be a high drop out rate considering that only 43% of the recruited patients were finally analysed. However to address the aims of the study it was necessary to have complete sets of data on exercise capacity and quality of life both at the beginning and at the end of OCR. Additionally we only included male patients.

The period of followup is quite short (12 weeks). Therefore we can not provide information about the longtime effects of the findings. Nonetheless we suppose that ongoing exercise training will preserve or even lead to a further improvement of sexual function.

The questions about sexual function have been default by the PLC questionnaire. In comparison to the UCLA Prostate Cancer Index Sexual Function Scale, for example, it is less extensive and has only the alternative of positive or negative answers but it covers mostly the same aspects of sexual function in a more general and less erection-centered way.

We did not analyse the reasons why patients did not complete the questionnaires. Therefore there might be a selection bias in parts of the patients considering that persons with sexual problems possibly did not want to answer questions on this topic.

Other patients might have had language problems and therefore were unable to answer the questions.

However the incidence of sexual dysfunction was similar to other studies, and therefore we think that this possible bias did not change the results significantly.

## Figures and Tables

**Table 1:** Patient characteristics at baseline.

characteristics	*n* = 896
Age	61.5 (±10)
Hypertension	54.7%
Hyperlipidemia	70.0%
Diabetes Mellitus	14.4%
Smoking	33.1%
Family history	34.6%
Betablockers	90.2%
Calcium Channel Blockers	5.6%
ACE-inhibitors	43.1%
AT-2-blockers	5.8%
Statins	83.70%
Diuretics	12.50%
Body mass index	26 (SD 3.3)
Left ventricular Ejection fraction (LVEF)	56% (SD 16.4)
PCI	65,1%
CABG	29,2%
Valvular surgery/other Indications for OCR	5,7%

CCB: calcium channel blockers, BMI: body mass index, LVEF: left ventricular ejection fraction, CABG: coronary artery bypass graft, OCR: outpatient cardiac rehabilitation, and SD: standard deviation.

**Table 2:** Exercise test results at baseline and at end of OCR (*n* = 896).

	before OCR	after OCR	*P-*value
Maximum capacity (Watt)	130 ± 36	160 ± 44	<.0001
% target exercise capacity	81 ± 19%	99 ± 23%	<.0001
Heart rate at rest (bpm)	75 ± 15	71 ± 13	<.0001
Heart rate at max. workload (bpm)	124 ± 20	131 ± 20	<.0001
Syst BP at rest (mm Hg)	116 ± 30	128 ± 22	<.0001
Diast BP at rest (mm Hg)	74 ± 20	80 ± 14	<.0001
Syst BP at max. Watt (mm Hg)	170 ± 35	186 ± 30	<.0001
Diast BP at max. Watt (mm Hg)	82 ± 17	88 ± 16	<.0001

**Table:** (a) Sexual activity and dysfunction in males as reported in questionnaires (*n* = 896).

	baseline	end of OCR	*P*-value
No problems with sexual activity	366 (40.8%)	346 (38.6%)	.048
Decrease in libido	302 (33.7%)	313 (34.9%)	.324
Exhaustion during intercourse	58 (6.5%)	61 (6.8%)	.710
Problems with sexual function			
(erectile dysfunction, no orgasm)	162 (18.1%)	203 (22.7%)	.0001
No sexual activity at all	191 (21.3%)	180 (20.1%)	.161

**Table:** (b) Changes in sexual function during OCR (*n* = 896).

After OCR	better	similar	worse
Problems with sexual function	36 (4.0%)	804 (89.7%)	56 (6.2%)
Decrease in libido	46 (5.1%)	793 (88.5%)	57 (6.4%)
Exhaustion during intercourse	13 (1.5%)	867 (96.8%)	16 (1.8%)
Erectile dysfunction/no orgasm	19 (2.1%)	817 (91.2%)	60 (6.7%)

**Table 4:** Univariate analysis of variables related to any problems with sexual activity after OCR.

	no problems	any problems	*P*-value
	(*n* = 346)	(*n* = 550)	
Age	58 ± 10	63 ± 10	.0001
Body mass index	26 ± 3	26 ± 3	.76
MaxWatt (before OCR)	131 ± 40	119 ± 35	.0001
MaxWatt (after OCR)	162 ± 49	146 ± 43	.0001
Watt-improvement	57%	48%	.006
Change in exercise cap.	31 ± 26	26 ± 24	.003
LVEF	56 ± 15%	55 ± 17	ns
PCI	68%	62%	.07
CABG	20%	31%	<.0001
Hypertension	48%	59%	.001
Hyperlipidemia	66%	72%	.02
Diabetes mellitus	10%	15%	.03
Smoking	31%	34%	.32
Family history	38%	32%	.07
Betablocker	11%	9%	.41
QoL scale:			
Subj. performance (before OCR)	2.33	2.12	<.0001
(after OCR)	3.00	2.62	<.0001
Ability to relax (before OCR)	2.75	2.50	<.0001
(after OCR)	3.00	2.87	<.0001
Positive mood (before OCR)	2.20	2.00	<.0001
(after OCR)	2.60	2.25	<.0001
Negative mood (before OCR)	3.12	2.87	<.0001
(after OCR)	3.5	3.25	<.0001
Ability to relate (before OCR)	2.66	2.5	<.0001
(after OCR)	3.00	2.83	<.0001
Affiliation (before OCR)	3.4	3.2	<.0001
(after OCR)	3.4	3.2	<.0001

QoL scales mentioned and explained in the methods section. Scale 4 (negative mood) is given as reciprocal value, that is, higher scores mean less negative mood.

**Table:** (a) Multivariate regression analysis of predictors of sexual dysfunction after OCR (without PLC). CABG: coronary artery bypass graft, ExC: exercise capacity.

	OR	95% confidence interval	*P*-value
Age per year	1.032	1.017–1.047	<.0001
Male gender	1.95	1.27–2.98	.002
CABG	1.39	1.01–1.92	.045
Hyperlipidemia	1.42	1.07–1.88	.014
Baseline ExC per Watt	99%	0.990–0.999	.01
Improvement of ExC per Watt	0.992	0.987–0.998	.004

**Table:** (b) Multivariate regression analysis of predictors of sexual dysfunction after OCR (including PLC). Abbreviations see Table 5(a).

	OR	95% confidence interval	*P*-value
age per year	1.053	1.039–1.067	<.0001
male gender	1.522	1.042–2.223	.03
CABG	1.372	1.0–1.88	.05
hyperlipidemia	1.38	1.036–1.839	.028
scale 1 of PLC	0.736	0.609–0.890	.002
scale 6 of PLC	0.553	0.443–0.691	<.0001
baseline ExC per Watt	0.998	0.993–1.002	.31
improvement of ExC per Watt	0.994	0.989–1.0	.055
